# A *Chan* Dietary Intervention Enhances Executive Functions and Anterior Cingulate Activity in Autism Spectrum Disorders: A Randomized Controlled Trial

**DOI:** 10.1155/2012/262136

**Published:** 2012-05-14

**Authors:** Agnes S. Chan, Sophia L. Sze, Yvonne M. Y. Han, Mei-chun Cheung

**Affiliations:** ^1^Neuropsychology Laboratory, Department of Psychology, The Chinese University of Hong Kong, Shatin, NT, Hong Kong; ^2^Integrative Neuropsychological Rehabilitation Center, The Chinese University of Hong Kong, Shatin, NT, Hong Kong; ^3^Henan Songshan Research Institute for Chanwuyi, Henan 452470, China; ^4^Department of Special Education and Counselling, The Hong Kong Institute of Education, Tai Po, Hong Kong; ^5^Institute of Textiles and Clothing, The Hong Kong Polytechnic University, Kowloon, Hong Kong

## Abstract

Executive dysfunctions have been found to be related to repetitive/disinhibited behaviors and social deficits in autism spectrum disorders (ASDs). This study aims to investigate the potential effect of a *Shaolin*-medicine-based dietary modification on improving executive functions and behavioral symptoms of ASD and exploring the possible underlying neurophysiological mechanisms. Twenty-four children with ASD were randomly assigned into the experimental (receiving dietary modification for one month) and the control (no modification) groups. Each child was assessed on his/her executive functions, behavioral problems based on parental ratings, and event-related electroencephalography (EEG) activity during a response-monitoring task before and after the one month. The experimental group demonstrated significantly improved mental flexibility and inhibitory control after the diet modification, which continued to have a large effect size within the low-functioning subgroup. Such improvements coincided with positive evaluations by their parents on social communication abilities and flexible inhibitory control of daily behaviors and significantly enhanced event-related EEG activity at the rostral and subgenual anterior cingulate cortex. In contrast, the control group did not show any significant improvements. These positive outcomes of a one-month dietary modification on children with ASD have implicated its potential clinical applicability for patients with executive function deficits.

## 1. Introduction

Executive dysfunction is a typical cognitive deficit associated with autism spectrum disorders (ASDs) which has been studied for over two decades. Executive functions refer to higher-order cognitive processes, such as working memory, attention, planning, response inhibition, mental flexibility, and self-monitoring. Well-balanced coordination among these various functions is crucial for the efficient execution of goal-directed behaviors. Although there is still debate on whether executive dysfunction really exists in ASD, it has been generally accepted in several recent reviews that deficits in planning, response inhibition, and mental flexibility are three relatively more common executive dysfunctions found in individuals with ASD [1]. Planning ability requires constant monitoring, evaluating, and updating of one's actions to formulate efficient strategies in problem solving. Impairment in planning and strategy formation are relatively prominent in children with ASD at younger ages (<11 years) [2] or with mental retardation [3]. Mental inflexibility is more commonly found in ASD individuals with lower intellectual functioning and verbal deficits when they are performing tasks that require flexible shifting of thought and action in response to situational changes [4]. In terms of response inhibition, that is, to execute inhibitory control of irrelevant or interfering information or impulses, individuals with ASD are more likely to demonstrate deficits when the tasks require inhibition of prepotent responses or when the inhibitory process entails more efforts that require additional working memory load [5]. The common ground for these three executive functions is a high demand on effective selection and initiation of context-appropriate responses and inhibition of inappropriate/irrelevant responses.

It has been suggested that the executive dysfunctions that involve inefficient and inappropriate selection, initiation, and suppression of thoughts and behaviors account for typical autistic features, such as the strong need for sameness, repetitive behaviors, restricted interests, uncontrollable behavioral and emotional reactions, and impaired social communication and interaction abilities. Some empirical studies have revealed a possible linkage between executive dysfunctions and autistic symptomatology [6–11]. For instance, Kenworthy and colleagues [6] reported significant relationships between both laboratory tasks testing executive functions and behavioral symptoms of ASD. Specifically, the performance of an inhibitory control test was negatively correlated with the severity level of restricted and repetitive behaviors in autism as measured by the Autism Diagnostic Interview and the Autism Diagnostic Observation Schedule. Some other studies [7–9] have also found a significant association between cognitive flexibility or set-shifting and repetitive behaviors in both children and adults with ASD. Another study further reported that perseverative responses in executive functioning tests on young autistic children were correlated with deficits in their social communication skills [10]. Berger et al. [11] found that performance in a cognitive flexibility task was a better predictor of social understanding and competence in autism.

Given that effective pharmacological intervention is not yet available for enhancing the executive functions of ASD, the majority of interventions that target the executive control of emotions and behaviors in autism are primarily behavioral or educational based [12, 13]. Although some behavioral interventions are found to be effective in eliciting context-appropriate behaviors and reducing problematic behaviors or emotional outbursts, the programs tend to be very intensive and time consuming [14, 15]. For instance, applied behavioral analysis is the most extensively researched educational-behavioral intervention and has been reported to show promising treatment effects on reducing various autistic symptoms. Yet, the most favorable outcomes are suggested to occur when the training programs are started before age 5 and intensively implemented for at least 20 hours per week for two or more years [15]. In addition, children with limited mental abilities who are not able to follow the rules and requirements of the training or have more severe autistic symptoms tend to show less positive outcomes from the many behavioral interventions [16]. Given the limitation of conventional intervention, some researchers have been studying various less conventional and exploratory methods, such as dietary interventions [17, 18], nutritional supplements [19, 20], music therapy [21–23], massage [24–26], and acupuncture [27, 28]. Although some preliminary data have revealed positive outcomes of these novel interventions, the treatment efficacy remains largely inconclusive [29]. The purpose of the present study is to explore a traditional Chinese concept of healing that has been practiced within the *Shaolin* temple for over a thousand years and examine its effect on reducing some of the executive dysfunctions in ASD.

 The *Shaolin* temple, although a Buddhism monastery, has been renowned for its martial arts and medicine. The concept of “food as medicine” has been well applied as clinical intervention for promoting mental and physical health within the *Shaolin* medical approach (*Chanyi*). It is believed that inappropriate intake of certain types of food may have harmful effects on physical health, mental state, and cognitive functions of human beings. For instance, the *Bencao Gangmu* written by *Li Shizhen* during the *Ming* Dynasty clearly stated that the long-term ingestion of garlic may cause forgetfulness, and excessive intake of ginger can be harmful to the mind and intelligence. The *Chanyi* proposed that excessive intake of hot and spicy foods (including all meats, seafood, eggs, ginger, garlic, spring onions, Chinese chives, and chili food) with high-fat and high-energy content will generate excessive heat inside the body and cause blood and *Qi* stagnation, which in turn, result in both physical and mental illnesses.

The *Chanyi* believed that the body can heal itself. In order to facilitate the self-healing process, it recommends the adoption of a natural and balanced vegetarian diet and reduction of the intake of the mentioned hot and spicy foods. The *Chanyi* (also termed as *Dejian* mind-body intervention) [30] has been examined recently in empirical studies and revealed positive results on improving physical health, mood and cognitive functions in community-dwelling adults [31], children with autistic/Asperger's disorder [32, 33], and individuals with brain damage [34] and depression [35, 36]. While previous studies have examined the effects of the holistic approach of *Chanyi* including psychoeducation, mind-body exercises, and diet modification, the present study aims to examine the effect of a single component within the model, that is, diet modification, as a possible intervention for children with ASD. Given the encouraging findings in previous studies, it is anticipated that a change in diet will reduce some of the executive function deficits (as measured by standardized neuropsychological tests) and related behavioral and social problems (as measured by parental reports on daily behaviors and social communication abilities) in children with ASD in an experimental group, as compared with their counterparts, the control group, who did not change their diet.

In addition, the present study will attempt to explore the possible neural mechanisms that underlie the potential treatment effects of diet modification by examining the pre-post alteration in neuroelectrophysiological responses of ASD children while performing a Go/No-go task. The Go/No-go task is selected because it is a common cognitive test that measures the executive control of response selection, execution, and inhibition, and some previous studies have found deficient performance in this task among ASD individuals [37, 38]. An extensive neural network that covers the prefrontal cortex, anterior cingulate cortex (ACC), and parietal lobes has been found to be activated during the Go/No-go task [38, 39]. Specifically, the ACC plays a major role in attentional control, error detection, response inhibition, and conflict monitoring [40–42]. Given that our recent study has revealed specific significant suppression in event-related theta activity of the ACC during a Go/No-go task in children with ASD [43], together with other repeated empirical evidence for hypoactive ACC in ASD [44–46], it is thus suggested that suppressed ACC activity may be a possible underlying factor for the impaired executive control of behaviors in the ASD population. Therefore, it is anticipated that children who have improved executive functions after dietary modification will demonstrate increased ACC activity during the Go/No-go task, whereas those without intervention will not show this increase.

## 2. Methods

### 2.1. Study Design

The study was designed as a randomized controlled trial. The recruited children with ASD were randomly and equally assigned into the experimental group (who were required to follow the *Chanyi* approach in diet modification) or the control group (who were reminded to maintain their regular usual diet). Measurements on various executive functions, behavioral problems, and event-related neurophysiological activities were obtained before and after the one month of changed/unchanged diet regime. 

### 2.2. Participants

Twenty-four children with ASD between the ages of 7 to 17 years old voluntarily participated in the study with written consent from their parents. The children were recruited from the database at the Neuropsychology Laboratory of the Chinese University of Hong Kong. All of the children received formal diagnosis of autistic disorder or pervasive developmental disorders, not otherwise specified by a clinical psychologist through a standard clinical interview with their parents based on the DSM-IV-TR criteria [47]. The degree of severity in autistic symptoms, including those that are related to social interaction, communication, and repetitive/stereotyped behaviors, was assessed by a clinical psychologist with the Autism Diagnostic Interview-Revised (ADI-R) [48]. The interview covers detailed questions about the early development and current functioning of the child, with higher scores indicating more severe autistic symptoms. Children with other neurodevelopmental, psychiatric, or neurological comorbidities or prescribed psychiatric medication were excluded from the study. 

The 24 children were randomly and equally assigned into the experimental (with diet modification) or the control group (without diet modification).  Table 1 presents the demographic and clinical characteristics of the two groups. Children in both groups were matched on age [*t*(22) = −1.296, *P* = 0.21], gender [*χ* ^2^(1) = 0.00, *P* = 1.00], and severity level of the autistic symptoms as measured by the four ADI-R subscales, *t* ranges from −1.064 to 0.822, and *P* ranges from 0.30 to 0.86. The two groups also demonstrated comparable levels of general intelligence [*t*(22) = 1.112, *P* = 0.28], which were assessed by the research assistant by using the short forms of the Chinese version of the Wechsler Intelligence Scale for Children-Third Edition (WISC-III) [49] or the Stanford-Binet Intelligence Scale-Fourth Edition (SB-FE) [50] for nonverbal children or those who showed a floor effect on the WISC-III. Among the 12 children in each group, 9 in the control group and 8 in the experimental group had limited intelligence with an IQ score at or below 70. 

### 2.3. Procedures

Prior to the baseline assessment, all of the children and their parents were briefed on the procedure of the assessments and informed consents from the parents were obtained. Then, the parents were interviewed by a clinical psychologist on their child's developmental and medical history based on a structured clinical interview. Meanwhile, the children were individually assessed by trained research assistants on their intellectual functioning, executive functions, and scalp electroencephalography (EEG) activities in a quiet room. The clinical psychologist and research assistants who conducted the assessments were blinded to the study design and group assignment. During the EEG recording, each child was required to perform a Go/No-go task while their EEG data were obtained by using a TruScan measuring set through 19 electrodes positioned across the scalp according to the International 10–20 System [51]. With electrode impedances maintained at ≤10 kΩ, the EEG signals were referenced to linked ears and sampled at 256 samples per second, with a high-frequency limit band pass of 30 Hz, and then fast Fourier transformed. Details of the Go/No-go task will be elaborated in a later section. Artifact-free EEG data were selected based on visual examination for eye movements and muscle artifacts and then captured for subsequent low-resolution electromagnetic tomography (LORETA) analyses. 

After the baseline assessments, the parents of the children in the experimental group were guided on a specific diet modification based on the *Chanyi* approach for one month, whereas parents of the children in the control group were recommended to maintain their usual dietary habits during the study period. After one month, the same assessments on the executive functions and EEG activity were performed on all of the children. Their parents were also requested to rate their child's executive-function-related behaviors (including social communication problems and repetitive/disinhibited behaviors) in daily life before and after the intervention.

### 2.4. Neuropsychological Assessments

Four major aspects of executive functions, including attention, mental flexibility, response inhibition, and planning, were measured by standardized neuropsychological tests before and after the intervention.

#### 2.4.1. D2 Test of Concentration (D2) [52]

This is a test on attention and inhibitory control that involves 14 lines of the letter “d” or “p” with a different number of dashes above and/or below the letter. The child was required to cancel as many “d”s with 2 dashes as possible within 20 seconds for each line, while ignoring the nontarget distractors. The concentration performance score which measures the degree of accuracy was used as a measure of attention. The number of commission errors (i.e., incorrect cancellation of distractors) was counted as a measure of disinhibition.

#### 2.4.2. Go/No-Go Task

This is a computerized task to test the ability to attend to and flexibly respond to changing stimuli and inhibit unwanted responses. A total of 192 black balls and 48 red balls (black : red ratio = 4 : 1) were randomly displayed, one at a time, for 500 ms followed by 1000 ms of blank intervals, in the center of a computer screen. The total testing time was 6 minutes. The child was required to press a key as quickly as possible in response to a black ball (Go stimulus), but to inhibit their response when a red ball (No-go stimulus) appeared. The omission errors at the Go condition were used as a measure of attention, whereas the commission errors during the No-go condition were used as an indicator of disinhibition.

#### 2.4.3. Children's Color Trails Test (CCTT) [53]

The second trial of the CCTT was adopted as a test of mental flexibility. It involves duplicates of each number embedded within pink and yellow circles and requires the child to connect the numbers in ascending order from 1 to 15 while alternating between the two colors as quickly as possible. The completion time was used as a measure of mental flexibility.

#### 2.4.4. The Five-Point Test (FPT) [54]

This figural fluency test was adopted as a test for mental flexibility, as it requires spontaneous generation of novel designs without repetition by connecting five points with straight lines within 5 minutes. A greater number of unique designs generated indicates higher flexibility.

#### 2.4.5. The Tower of California (ToC) Test [55]

The ToC was adopted as a test of planning ability, strategy formulation, and inhibition. It consists of nine items that involve moving discs on three colored vertical pegs to match a target arrangement while adhering to rules. The total achievement score, which is composed of accurate and efficient movements and successful completion of items, was calculated as a measure of planning.

### 2.5. Parental Evaluation on Behavioral Measures

Given that executive dysfunctions in autism was found to be associated with repetitive/disinhibitory behaviors and social communication abilities, the behavioral changes in ASD children in the present study were examined based on parental evaluation. Their verbal and nonverbal social communication abilities were measured by two subscales from the Autism Treatment Evaluation Checklist (ATEC) [56], namely, communication and sociability, on a 3-point scale (from “0” to “2”), in which a higher score indicates greater problems. The parents were asked to rate a total of 14 items in the communication subscale and 20 items in the sociability subscale before and after the one-month period. The total scores of the two subscales were averaged for a pre-post comparison of each group. For repetitive/disinhibitory behaviors, the parents were asked to rate based on a questionnaire composed of 7 questions that measured the problems of controlling repetitive speech/acts, rigid thoughts/acts, and hyperactive behaviors of their child. They were required to evaluate the degree of change in each behavior problem of their child after one month by using a scale of “−5” to “+5”, where “+5” indicates “largely improved”, “−5” indicates “largely declined,” and “0” indicates “no change”. The average score obtained was used for subsequent comparisons. 

### 2.6. EEG Assessments

The EEG data of each child was first transformed by Excel application before imported into EEGLAB software by using MatLab 7.1 to capture the correct events and epochs. The epoch limit was set as 50 ms as the start and 900 ms as the end. Artifacts in epoched data were then pruned by visual inspection and by using the rejection method on the EEG Plot. All incorrect hits were also deselected. The transformed data were exported and then spectrally processed by using fast Fourier transformation (FFT) to compute the power data for the theta band (4–7.5 Hz) through the use of NeuroGuide software. Given that the ACC has been reported as one of the generators for theta activities in the human brain [57–59], LORETA [60, 61] was adopted to localize the sources of the theta activities in response to the “Go” and ‘‘No-go” conditions. The sources of the theta activities were expressed as three-dimensional cortical current density according to the Talairach brain atlas. 

### 2.7. Intervention: Specific Diet Modification

Children in the experimental group were recommended to reduce their intake of some foods (including ginger, garlic, green onion, spicy foods, eggs, meat, and fish) which will generate excessive internal heat and adversely affect the temper and cognitive functions. It should be stressed that the children were not required to abstain from these foods, but advised to cut down on their intake according to their own lifestyles and plans. The parents were advised to gradually change their child's diet and required to complete dietary log sheets to verify and monitor their progress weekly. To ensure a well-balanced nutritional diet, the children in the experimental group were also encouraged to take food from the seven categories every day with one to three kinds from each category. The seven categories are (1) grains (e.g., noodles, brown rice); (2) vegetables (e.g., broccoli, tomatoes); (3) fruits (e.g., grapes, apples); (4) beans (e.g., soy, peas); (5) mushrooms (e.g., black fungus, straw mushrooms); (6) nuts (e.g., walnuts, almonds); (7) roots (e.g., potatoes, yams). The type and amount of food were not specified, as long as they were fresh and seasonal and the children ate until they were 80% full for each meal. The log record revealed that 33% of the children completely followed the dietary recommendation, that is, reducing intake of or abstaining from all hot and spicy foods and consuming from all the above seven recommended food categories. Eighty-nine percent of the children reduced or abstained from consumption of ginger, garlic, green onion, and spicy foods, and 78% had some meat, fish, and egg throughout the one-month period. The control group received no dietary recommendation and was reminded to maintain the same dietary habits throughout the study. 

### 2.8. Data Analyses

The performance on various neuropsychological measures on executive functions before and after the one month was compared for each group of children by using repeated measures ANOVA and then followed by *post hoc* paired *t*-tests. The corresponding effect sizes were also computed for comparison between groups. Within- and between-group comparisons on parental rating of changes in the behaviors of their children after one month were also performed using paired and independent sample *t*-tests. To examine the effect of diet modification on neural activity, LORETA voxel-by-voxel paired-sample *t*-tests were performed on EEG data with subjectwise normalization and log transformation for each group of children in comparing the pre-post changes in sources of theta activity at the ACC during the “Go” and ‘‘No-go” conditions. Given that specific hypotheses were tested and the number of participants was relatively small, no adjustment to the alpha level was applied to avoid lowering the power of the tests. The effect size was provided to evaluate the extent of the effect of the treatment.

## 3. Results

### 3.1. Diet Change Improved Some of the Executive Functions in ASD Children

At the baseline, the control and the experimental groups showed comparable levels of functioning in attention, mental flexibility, response inhibition, and planning as measured by the 7 neuropsychological measures, *t*s range = −0.91 to 1.26, *P* > 0.05, without significant differences between the two groups in any of the measures.  Table 2 presents the mean performance of each group on various executive functioning tests before and after the one-month period. The results of the repeated measures ANOVAs showed significant time (pre versus post) by group (experimental versus control) interaction effect on one flexibility measure only (FPT: *F* = 8.06, *P* = 0.01) and significant main effect of time on an attention measure (D2-concentration performance: *F* = 5.04, *P* = 0.04) and another flexibility measure (CCTT: *F* = 9.29, *P* = 0.01). Given that the nonsignificant multivariate results may be possibly due to large within-group variations (as revealed in Table 2 in which the standard deviations in the majority of the measures are approaching or even greater than the group mean), paired-sample *t*-tests were thus performed to explore the trend of pre-post differences in each group. After one-month modification in diet, the experimental group showed significantly improved performance in the three executive function domains, including mental flexibility (FPT: *t* = 3.614, *P* = 0.004; CCTT: *t* = −2.647, *P* = 0.017), response inhibition (D2: *t* = −2.771, *P* = 0.016), and planning (ToC: *t* = 2.072, *P* = 0.039). The effect size of these three measures on mental flexibility and inhibitory control with positive treatment effects was large (0.94 to 1.20), and the planning measure had a medium effect size (0.73). The extent of improvement in the performance of the experimental group ranged from 37% to 62%. In contrast, the control group did not show significant improvement in any of the domains, *t*s range = −0.42 to 1.59, *P* > 0.05, effect size = 0.03 to 0.56, with the extent of improvement ranging from 3% to 29%. In summary, diet modification based on the *Chanyi *approach for one month has positive effects on improving mental flexibility, response inhibition, and planning in children with autism. 

For the measure of attention ability, both the experimental and control groups demonstrated a trend of improvement in the concentration performance score of the D2 test with a medium effect size (0.69 and 0.54), *t* = 1.83 and 1.60, *P* = 0.059 and 0.074, respectively. The experimental group also showed a reduced number of omission errors in the Go/No-go task (from 38.0 to 31.8) with a small effect size of 0.42, although this was not significant, *t* = −1.33, *P* = 0.109. However, the control group had more omissions after one month (from 20.2 to 25.0), *t* = 1.01, *P* = 0.170, although this was not significant. It seems that diet modification has a smaller extent of positive effects on the attention ability of ASD children. 

Given that diet modification has significant positive effects on the four measures of mental flexibility, inhibitory control, and planning, pre-post analyses on these four measures were performed on the low-functioning subgroups (i.e., IQ ≤ 70) to explore if a similar treatment effect is replicable on them. There were 8 and 9 low-functioning children in the experimental and control groups, respectively. The results of paired *t*-tests showed that a similar extent of improvement in all three measures of flexibility and inhibition was replicable in the experimental low-functioning subgroup (with a medium to large effect size), and the control subgroup consistently showed no significant improvement in any of the measures (Table 3). Even with the small sample size, the performance of the two measures on mental flexibility and response inhibition remained statistically significant with a large effect size (CCTT-T2: time: *t* = −2.89, *P* = 0.032; D2: commission: *t* = −2.97, *P* = 0.049). For the performance change in the ToC (the planning test) of the experimental group, the effect size was reduced from previously medium (0.73) in the analysis for the entire group, to small (0.43) in the subgroup analysis. This finding has provided preliminary support for the positive effect of diet change in mental flexibility and response inhibition of low-functioning ASD children. 

### 3.2. Parental Evaluation on Daily Behavioral Control and Social Communication after Diet Modification

**Figure 1:**
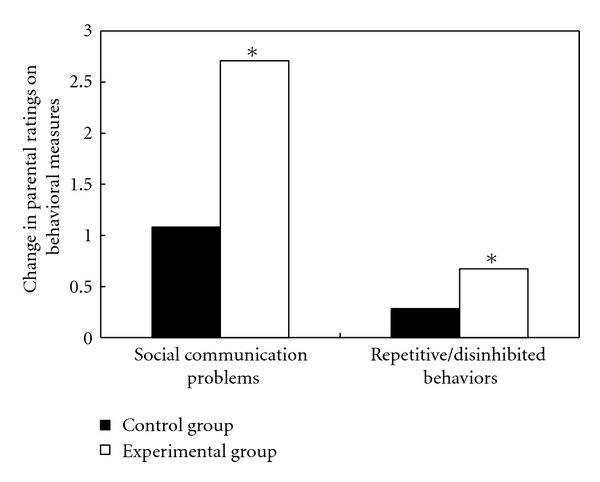
Changes in parental ratings on social communication problems and repetitive/disinhibited behaviors of their children after a one-month period. A positive value indicates improvement in the corresponding problem. *P < 0.05 (paired-sample *t*-test or one sample *t*-test).

In terms of the average score of the pre-post comparison for the ATEC communication and sociability subscales, the evaluations of parents with children in the experimental group indicated that they significantly demonstrated fewer social communication problems after the diet change for one month, *t*(11) = 3.898, *P* = 0.001, effect size = 1.12. Such significant improvement was not reported by parents of the control group children, *t*(11) = 1.324, *P* = 0.106, effect size = 0.38 (Figure 1). The experimental group demonstrated a 21% reduction in problematic social behaviors, which was twice that of the control group (11%). 

In addition, parents of the children in the experimental group reported a significantly positive change (mean rating = 0.67) in the executive control of repetitive, inflexible, and hyperactive behaviors of their children after intervention, *t*(11) = 1.871, *P* = 0.044, effect size = 0.54, whereas children in the control group were not reported to have any significant behavioral changes (mean rating = 0.28) by their parents, *t*(11) = 1.011, *P* = 0.167, effect size = 0.29 (Figure 1). While 58% of the children in the experimental group received improved ratings, 25% of their counterparts in the control group received improved ratings, but 17% were evaluated to have experienced deterioration. This suggests that diet modification was considered by the parents of ASD children to have some positive effects on improving their child's daily behavioral control and social communication abilities. 

### 3.3. Diet Change Enhanced Theta Activity in Anterior Cingulate Cortex of ASD Children

**Figure 2:**
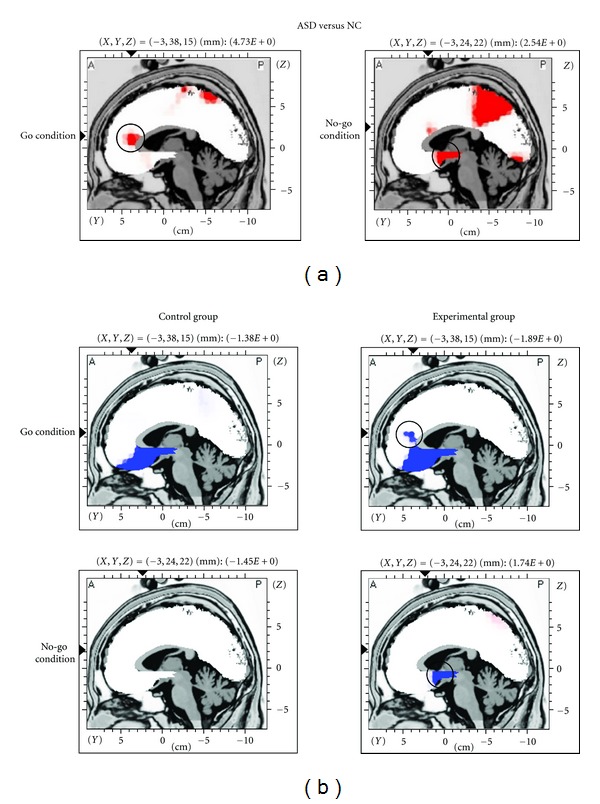
(a) Graphical representation of LORETA independent *t*-statistics results adopted from the research article by Chan et al. [43] published in the Research in Autism Spectrum Disorders. The location of the voxel is defined by the Talairach coordinates (*X*, *Y*, *Z*), and the regions colored in red represent significant hypoactivity in the anterior cingulate cortex (ACC, in the circle) of children with autism spectrum disorders (ASDs) as compared to their normal counterparts (NCs) during the Go and No-go conditions. (b) Graphical representation of LORETA paired *t*-statistics results that compares the pre- and post-one-month theta source activity of the control and experimental groups during the Go and No-go conditions. The regions colored in blue indicate significantly elevated ACC activity after one month at *P* (one-tailed) < 0.05. The ACC regions (in the circle), in which hypoactivity was previously found in children with autism spectrum disorders, were enhanced in activity after diet modification in the experimental group.

 Given that the effect of diet modification seems to be more pronounced in the flexible inhibitory control of responses in ASD, further pre-post comparisons on EEG activities during the Go/No-go task (a common test of inhibitory control) were performed. LORETA voxel-by-voxel paired *t* statistics were performed for each group of children during the Go and No-go conditions to examine the effect of diet modification in enhancing activity level in the ACC. The ACC was selected as the region of interest because our previous study has revealed hypoactive ACC patterns in ASD children in both Go and No-go conditions, as compared to their normal counterparts [43] (circled regions in Figure 2(a)). 

At the baseline, the voxel-by-voxel independent *t* statistics showed that both the control and experimental groups have comparable activity levels in the ACC at both the Go and No-go conditions, *P* = 0.538 and 0.609, respectively. After one month of change in diet, the experimental group showed significantly elevated activity specifically at the rostral ACC regions (Brodmann areas 24 and 32) during the Go condition, maximal *t* = 1.89, *P* < 0.05 (circled region in the upper image of Figure 2(b)). This specific region with increased activity after the diet change was the region that showed hypoactivity in the ASD children of our previous study (circled region in the upper image of Figure 2(a)). In contrast, the control group did not show significant activity change in the rostral ACC, maximal *t* = 1.25, *P* > 0.05. During the No-go condition, the experimental group showed significantly increased activity in the subgenual ACC region (Brodmann area 25) and subcallosal gyrus after one-month intervention (circled region in the lower image of Figure 2(b)), maximal *t* = 2.04, *P* < 0.05, which were also the regions that showed hypoactivity in ASD children in our previous study (circled region in the lower image of Figure 2(a)). For the control group, there was no significant change in ACC activity after one month, maximal *t* = 0.24, *P* > 0.05. 

## 4. Discussion

 Typical symptoms of ASD, including repetitive mannerisms, impulsive acts, emotional outbursts, restricted interests, inflexible adherence to specific routines, and social communication deficits, were found to be correlated with executive dysfunctions in response selection, alteration, and inhibition. Intervention that can reduce the executive dysfunctions of children with ASD may reduce their behavioral problems in daily life. The present findings showed that a specific diet modification based on the Chinese *Chan* medical approach had some positive effects on improving executive functions and typical behavioral symptoms of children with ASD. These results are encouraging since commonly used behavioral interventions for ASD children are very time consuming and not cost-effective, while the present diet modification is less time consuming and more economical. 

 The present finding may suggest an alternative or complementary intervention for the executive control of behaviors among ASD children. It should be noted that the positive effect of diet modification was also applicable to the low-functioning children with IQ at or below 70 in this experiment. Specifically, there were 8 low-functioning children in the experimental group and 9 in the control group. Those in the experimental group showed improvement in tests of mental flexibility and inhibitory control with a large effect size (although some pre-post differences did not reach significance due to the small sample size). However, the low-functioning children in the control group did not show such improvement. Given that behavioral training is particularly difficult and time and labor intensive when applied on children with limited intelligence, diet modification that can be monitored by parents may therefore be considered as an alternative. 

The underlying mechanism that may explain the change in behaviors was evaluated with an electrophysiological method. LORETA localization of theta activity showed that after one month of diet modification, the ASD children demonstrated increased activation of the ACC specific to the rostral and subgenual subdivisions. The control group did not show such improvement. Our previous study found that ASD children demonstrated hypoactivity in the ACC when performing a response-monitoring and inhibitory task (i.e., Go/No-go task) as compared to normally developed children [43]. The present study showed that a one-month diet modification was able to enhance the activity of the ACC while the child was performing the same task. This finding suggests that the behavioral improvement of children with ASD may be associated with increased activity in the ACC; however, how diet can change and improve activity in the neural system warrants further investigation in future studies. 

The therapeutic effects of diet modification on brain functions and activity in the present study are consistent with our decades of clinical observations and some empirical findings in past years. For instance, two studies have found significantly enhanced frontal activity and improved attention in community-dwelling adults with depressed mood and patients with clinical depression after receiving the *Dejian* mind-body intervention with diet modification as one of the treatment components [31, 35, 36]. Another case study on a low-functioning autistic child also demonstrated significantly improved inhibitory control and cognitive flexibility and increased EEG cordance (an index associated with cerebral perfusion) of the whole brain after *Dejian* mind-body intervention [32]. Thus, the present study has shed some light on the potential application of the Chinese *Chan*-based diet modification as a complementary intervention for rehabilitation of individuals with ASD and other individuals with emotional and cognitive problems. 

The idea of food as medicine has also drawn increasing attention in western scientific research. A number of studies have supported the beneficial effects of a vegetarian or vegan diet in promoting the health of cardiovascular and digestive systems, reducing cancers and degenerative diseases and improving mood [62–64]. Some studies have also revealed a significant linkage between a balanced nutritional diet and level of cognitive functions and cognitive development in early life [65, 66]. However, many of them are observational studies and there is also counterevidence against the positive dietary effects [67]. Therefore, it remains inconclusive in terms of the actual outcomes and the choice of type of diet. 

The present study has shown the positive effects of a one-month dietary modification on the executive functions and autistic symptoms of children with autism; however, its long-term effect is still unknown which is worth further investigation. In addition, the sample size of the low-functioning subgroup is relatively small; therefore, future studies with larger sample sizes will be helpful to verify the effect of diet modification. Given the preliminary evidence on the effects of diet change in the low-functioning subgroups, it will also be worth investigating if this can benefit patients with severe brain disorders or physical disabilities (e.g., demented or stroke patients) in a well-controlled study. Last but not least, given the increasing research interest in food as medicine to improve health in Western countries, the applicability and effectiveness of this Chinese *Chan*-based dietary modification to the Caucasian population can also be investigated in future studies. 

## 5. Conclusion

This study has provided some evidence for the therapeutic effect of a Chinese *Chan*-medicine-based diet modification carried out in the time span of one month on improving executive functions, reducing autistic symptoms and enhancing the ACC activity of children with ASD. Such encouraging findings have shed some light on the potential applicability of this specific diet modification for people who suffer from executive function deficits. 

## Figures and Tables

**Table 1:** Baseline demographic and clinical characteristics of participants in the control and experimental groups.

Characteristics	Control group (*n* = 12)	Experimental group (*n* = 12)	*t*/*χ* ^2^	*P* value
Age (mean ± SD), years	12.33 ± 2.38	11.06 ± 2.43	−1.30	0.21
Gender, male (%)	83.3	83.3	0.00	1.00
IQ (mean ± SD)	60.92 ± 20.83	73.50 ± 33.20	1.11	0.28
Diagnosis			0.17	0.68
Autistic disorder (%)	50	58.3		
PDD-NOS (%)	50	41.7		
Severity of disorder (mean ± SD)				
ADI-R social interaction	21.50 ± 5.42	21.00 ± 8.41	−0.17	0.86
ADI-R communication	14.08 ± 6.16	13.42 ± 3.99	−0.32	0.76
ADI-R stereotyped behavior	4.75 ± 2.77	3.42 ± 3.34	−1.06	0.30
ADI-R abnormal < 36 months	3.50 ± 1.00	3.92 ± 1.44	0.82	0.42

ADI-R: Autism Diagnostic Interview-Revised; IQ: intelligence quotient as assessed by the Chinese version of  Wechsler Intelligence Scale for Children-Third Edition or the Stanford-Binet Intelligence Scale-Fourth Edition; PDD-NOS: Pervasive Developmental Disorders, not otherwise specified.

**Table 2:** Mean performance in executive functioning of the control and experimental groups at before and after one-month period.

	Control group (*n* = 12)		*P* value	Effect size	Experimental group (*n* = 12)		*P* value	Effect size
	Before	After			Before	After		
Attention								
D2: concentration performance	78.67 (41.33)	98.22 (61.23)	0.074	0.535^+^	92.86 (50.47)	109.14 (53.00)	0.059	0.690^+^
Go/No-go: omission^#^	20.20 (22.08)	25.00 (30.67)	0.170	0.319	38.00 (43.06)	31.80 (38.32)	0.109	0.421
Mental flexibility								
FPT: unique design	15.67 (9.29)	16.22 (8.29)	0.320	0.137	12.56 (11.36)	20.33 (15.84)	0.004**	1.204^++^
CCTT-T2: time (in seconds)^#^	94.09 (72.17)	67.18 (32.64)	0.078	0.562^+^	132.08 (98.00)	78.73 (51.44)	0.017*	0.936^++^
Response inhibition								
D2: commission^#^	22.00 (27.24)	21.44 (29.05)	0.461	0.034	23.57 (38.21)	14.86 (32.32)	0.016*	1.047^++^
Go/No-go: commission^#^	13.00 (11.30)	11.40 (7.40)	0.216	0.261	13.27 (11.47)	15.27 (13.66)	0.204	0.260
Planning								
ToC: achievement score	5.70 (2.41)	6.20 (3.97)	0.344	0.131	6.25 (3.41)	8.88 (5.28)	0.039*	0.733^+^

FPT: five-point test; D2: D2 Test of Attention; CCTT-T2: time: total completion time in trial 2 of the Children's Color Trails Test; ToC: Tower of California Test. Standard deviations are in parenthesis. ^#^Lower value indicates better performance; *P < 0.05, **P < 0.01; ^+^medium effect size, ^++^large effect size.

**Table 3:** Mean performance in executive functioning of the control and experimental low-functioning subgroups at before and after one-month period.

	Control Group (*n* = 9)		*P* value	Effect size	Experimental Group (*n* = 8)		*P* value	Effect size
	Before	After			Before	After		
Mental Flexibility								
FPT: unique design	11.00 (6.99)	11.67 (4.80)	0.353	0.163	4.60 (4.34)	8.80 (6.18)	0.084	0.752^+^
CCTT-T2: time^#^	112.34 (89.16)	71.88 (41.88)	0.098	0.695^+^	184.25 (98.48)	110.27 (56.28)	0.032*	1.448^++^
Response Inhibition								
D2: commission^#^	26.00 (33.11)	23.67 (34.06)	0.384	0.128	43.33 (56.01)	31.33 (49.07)	0.049*	1.710^++^
Planning								
ToC: achievement score	5.29 (2.43)	4.86 (3.34)	0.309	0.199	3.75 (2.36)	5.75 (4.27)	0.228	0.426

FPT: five-point test; D2: D2 Test of Attention; CCTT-T2: time: total completion time in trial 2 of the Children's Color Trails Test; ToC: Tower of California Test. Standard deviations are in parenthesis. ^#^Lower value indicates better performance; *P < 0.05; ^+^medium effect size, ^++^large effect size.
